# The Protective Role of Apelin in the Early Stages of Diabetic Retinopathy

**DOI:** 10.3390/ijms232314680

**Published:** 2022-11-24

**Authors:** Jing Feng, Weiqiang Yang, Fuxiao Luan, Fang Ma, Yingjie Wang, Yiquan Zhang, Xuhui Liu, Li Chen, Xiaofeng Hu, Yong Tao

**Affiliations:** Department of Ophthalmology, Beijing Chaoyang Hospital, Capital Medical University, Beijing 100020, China

**Keywords:** apelin, pericytes, vascular leakage, early stages of diabetic retinopathy, protective role

## Abstract

Diabetic retinopathy (DR) is one of the most common and serious microvascular complications of diabetes. Although current treatments can control the progression of DR to a certain extent, there is no effective treatment for early DR. Apart from vascular endothelial growth factor, it has been noted that the apelin/APJ system contributes to the pathogenesis of DR. We used a high-fat diet/streptozotocin-induced type 2 diabetic mouse model. The mice were divided into a lentivirus control group (LV-EGFP), an apelin-overexpression group (LV-Apelin+), and an apelin-knockdown group (LV-Apelin−), all of which were administrated intravitreal injections. LV-Apelin+ ameliorated the loss of pericytes in DR mice, whereas LV-Apelin− aggravated the loss of pericytes. Similarly, LV-Apelin+ reduced the leakage of retinal vessels, whereas LV-Apelin− exacerbated it. The genes and signaling pathway related to cell adhesion molecules were downregulated, whereas the cell–cell tight junctions and anti-apoptotic genes were upregulated in response to apelin overexpression. However, the alterations of these same genes and signaling pathways were reversed in the case of apelin knockdown. Additionally, LV-Apelin+ increased ZO-1 and occludin levels, whereas LV-Apelin− decreased them. Our results suggest that apelin can reduce vascular leakage by protecting pericytes, which offers a promising new direction for the early treatment of DR.

## 1. Introduction

Diabetic retinopathy (DR) is one of the most common and serious microvascular complications of diabetes and the leading cause of blindness in people aged 20–70 years [1,2]. Although the anti-vascular endothelial growth factor (VEGF), laser photocoagulation, and vitrectomy treatments can control the progression of DR to a certain extent, there is no effective treatment for early DR.

Existing studies have indicated that early non-proliferative DR changes are due to pericyte loss, which results in the disturbance of retinal vascular microcirculation [3]. Pericytes are branch cells located on the capillary wall that are embedded in the microvascular basement membrane and wrap vascular endothelial cells to establish stable and strong physical connections [4]. In DR, there is a lack of pericytes, even though the endothelial cells of capillaries are intact [5]. Hyperglycemia-induced pericyte loss leads to increased vascular permeability and endothelial cell damage, which causes increased leakage, infiltration of immune cells, and development of proliferative DR [6]. 

In addition to VEGF, the apelin/APJ system has been noted to contribute to the pathogenesis of DR [7,8]. Except for its neovascularization effect in late-stage of DR, numerous experimental studies, including our previous study, have demonstrated that apelin can promote the development of normal blood vessels in the retina [9,10,11]. In an in vitro study, we also found that apelin could reduce pericyte apoptosis under hypoxic stimulation [12]. Apelin has also been proven to enhance endothelial cell–cell junctions to create non-leaky blood vessels [13].

As apelin has been recognized as a protective factor with regard to pericytes and endothelial cell–cell junctions, but has not been investigated in vivo thus far, we conducted this study to investigate the potential protective effects of apelin on pericytes and the blood-retinal barrier in the early stage of DR in vivo.

## 2. Results

### 2.1. Construction of an High-Fat Diet/Streptozotocin (HFD/STZ)-Induced Type 2 Diabetic Mouse Model and Various Lentiviral Vectors

**Figure 1:**
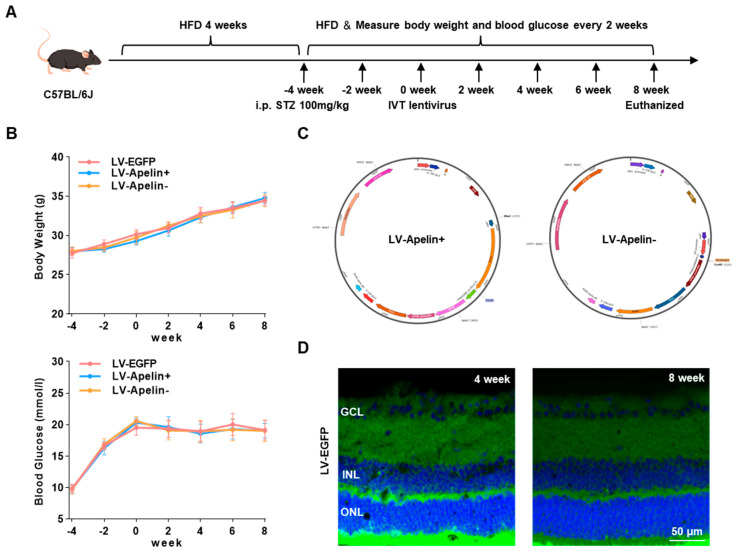
Construction of an HFD/STZ-induced type 2 diabetic mouse model and different lentiviral vectors. (**A**) Experimental and dosing procedures for HFD/STZ-induced type 2 diabetes. (**B**) Body weight and blood glucose were measured every two weeks. Data are expressed as mean ± SD (n = 6). (**C**) The maps of LV-Apelin+ and LV-Apelin−. (**D**) The expression images of LV-EGFP were obtained through confocal laser scanning microscopy (CLSM) at four and eight weeks. Blue: DAPI, green: LV-EGFP. GCL: ganglion cell layer; INL: inner nuclear layer; ONL: outer nuclear layer.

Figure 1A presented the complete experimental and dosing procedures for the HFD/STZ-induced type 2 diabetic mouse model. In this model, the mice gradually gained weight due to the HFD being continued until the end of the study, with their blood glucose rising to 16.7 mmol/L two weeks after STZ injection (Figure 1B). The maps of LV-Apelin+ and LV-Apelin− (Figure 1C) revealed that we successfully constructed the lentiviral vector with apelin overexpression and knockdown. Four and eight weeks after the intravitreal injection of LV-EGFP, the retinal sections revealed extensive LV expression in the retina, including the ganglion cell layer, inner nuclear layer, and outer nuclear layer (Figure 1D).

### 2.2. The Expression of Apelin in DR Mice Treated with LV-EGFP, LV-Apelin+ or LV-Apelin−

**Figure 2:**
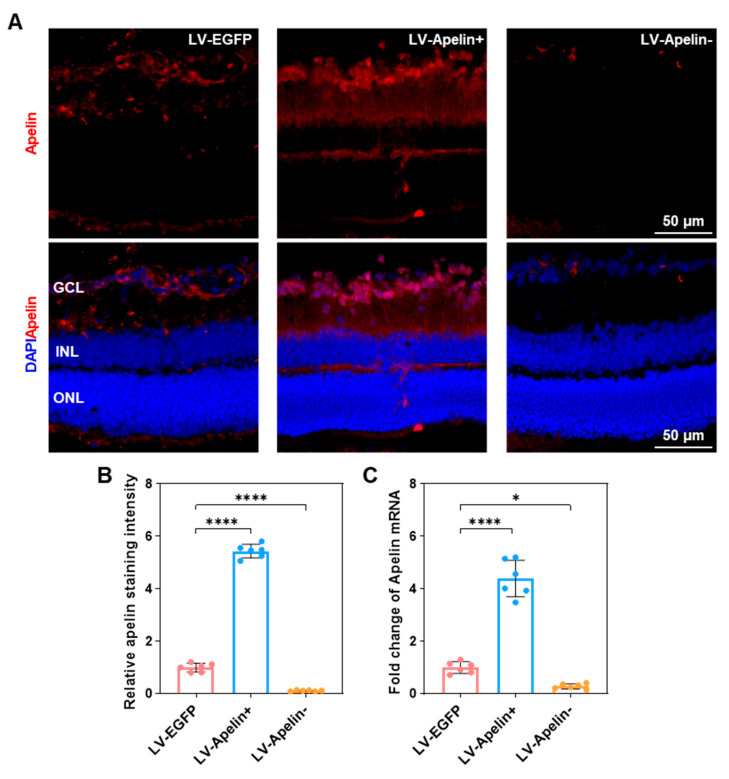
The expression of apelin in DR mice treated with LV-EGFP, LV-Apelin+, or LV-Apelin−. (**A**) Representative apelin staining images by CLSM and (**B**) the quantification of the staining intensity indicated that apelin was moderately expressed in the LV-EGFP group and was primarily distributed in the GCL, while it was widely expressed in the LV-Apelin+ group and distributed in the GCL and INL. In the LV-Apelin− group, there was almost no expression of apelin. (**C**) The qRT-PCR results revealed that the mRNA expression of apelin was upregulated in the LV-Apelin+ group, but downregulated in the LV-Apelin− group. Blue: DAPI, red: apelin. GCL: ganglion cell layer; INL: inner nuclear layer; ONL: outer nuclear layer. Data were expressed as mean ± SD. LV-Apelin+ versus LV-EGFP; LV-Apelin− versus LV-EGFP; * *p* < 0.05, **** *p* < 0.0001 (n = 6).

Immunofluorescence images (Figure 2A) and quantitative statistics (Figure 2B) of apelin indicated that compared to the LV-EGFP group, there was an increased expression of apelin when administering LV-Apelin+ and decreased expression of apelin when administering LV-Apelin−. Simultaneously, the expression of apelin mRNA (Figure 2C) was four-fold higher in the LV-Apelin+ group than that in the LV-EGFP group, and that in the LV-Apelin− group was one third of that in LV-EGFP group. These results demonstrated the high efficiency of gene overexpression and knockdown using a lentivirus vector.

### 2.3. The Expression of Pericytes in DR Mice Treated with LV-EGFP, LV-Apelin+, or LV-Apelin−

**Figure 3:**
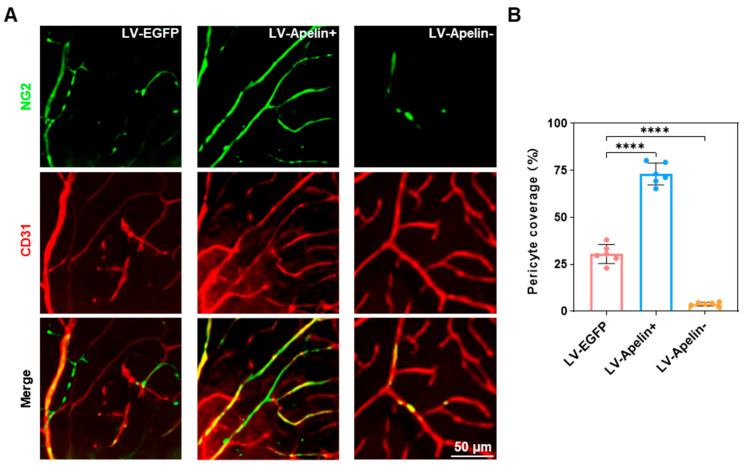
The expression of pericytes in DR mice treated with LV-EGFP, LV-Apelin+, or LV-Apelin−. (**A**) Representative NG2 and CD31 staining images by CLSM and (**B**) the quantification of pericyte coverage indicated that there was a partial loss of pericytes in the LV-EGFP group, while the expression of pericytes increased in the LV-Apelin+ group but decreased in the LV-Apelin− group. Green: NG2, Red: CD31. Data were expressed as mean ± SD. LV-Apelin+ versus LV-EGFP; LV-Apelin− versus LV-EGFP; **** *p* < 0.0001 (n = 6).

Since a previous study has revealed that NG2 is a biomarker specific to pericytes [5], we have also considered it an indicator of pericyte expression. Retinal whole-mount staining (Figure 3A) revealed that the overexpression of apelin decreased the loss of pericyte, whereas the knockdown of apelin increased the loss of pericyte. Furthermore, the corresponding quantitative analysis (Figure 3B) revealed that the coverage of pericytes increased from 30 percent to 70 percent in the LV-Apelin+ group and decreased from 30 percent to 5 percent in the LV-Apelin− group.

### 2.4. Retinal Leakage in DR Mice Treated with LV-EGFP, LV-Apelin+, or LV-Apelin−

**Figure 4:**
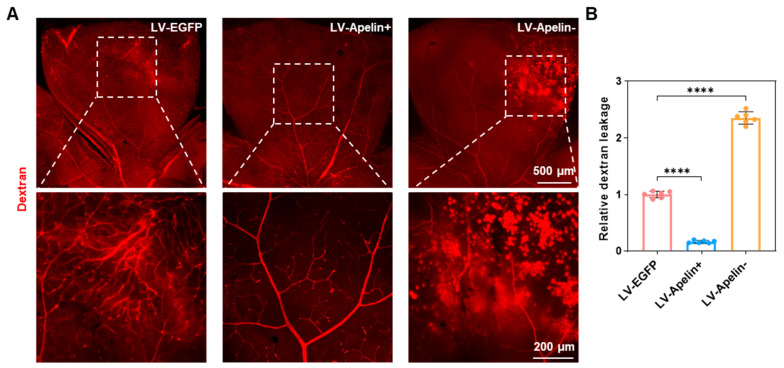
Retinal leakage in DR mice treated with LV-EGFP, LV-Apelin+, or LV-Apelin−. (**A**) Representative dextran leakage images and (**B**) the quantification of dextran leakage revealed that the leakage of retinal vessels was moderate in the LV-EGFP group; however, it decreased in the LV-Apelin+ group and increased in the LV-Apelin− group. Data were expressed as mean ± SD. LV-Apelin+ versus LV-EGFP; LV-Apelin− versus LV-EGFP; **** *p* < 0.0001 (n = 6).

Dextran is a polysaccharide that can be effectively used to assess the level of retinal vascular leakage [9]. Therefore, we used tetramethylrhodamine isothiocyanate–dextran to measure retinal leakage, which was directly observed via confocal fluorescence imaging. Figure 4A indicated that there was almost no retinal leakage in the LV-Apelin+ group; however, retinal leakage significantly increased in the LV-Apelin− group compared to that in the LV-EGFP group. Quantitative statistics (Figure 4B) indicated that LV-Apelin+ caused a 0.2-fold reduction in retinal leakage, and that LV-Apelin− caused a 2.4-fold increase in retinal leakage.

### 2.5. The Mechanism Underlying the Protective Role of Apelin in the Early Stages of Diabetic Retinopathy

**Figure 5:**
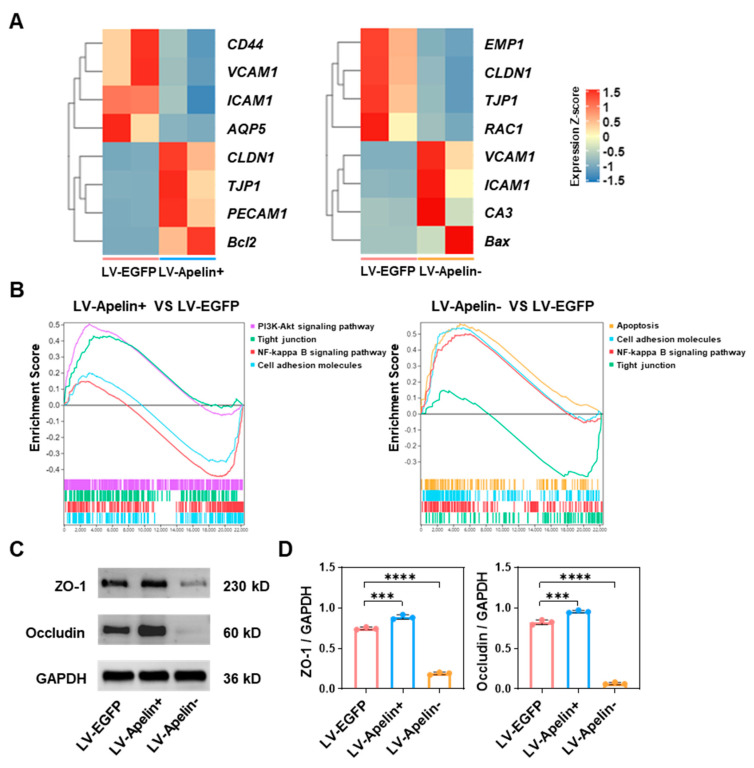
The mechanism underlying the protective role of apelin in the early stages of diabetic retinopathy. (**A**) The heatmap of differential gene expression showed that LV-Apelin+ upregulated genes related to tight junction, and anti-apoptosis downregulated genes related to the adhesion molecule and vascular permeability. In contrast, LV-Apelin− upregulated genes related to adhesion molecules and pericyte apoptosis, and downregulated genes related to tight junction genes (n = 2). (**B**) The results of GSEA were consistent with the differential gene expression analysis. (**C**) Representative Western blot images and (**D**) the quantification of the relative protein levels revealed that the levels of ZO-1 and occludin were increased by LV-Apelin+ but decreased by LV-Apelin−. The data were expressed as mean ± SD. LV-Apelin+ versus LV-EGFP; LV-Apelin− versus LV-EGFP; *** *p* < 0.001, **** *p* < 0.0001 (n = 3).

To elucidate the mechanism underlying the protective effect of apelin on pericytes and in reducing the retinal leakage of apelin, we conducted the RNA sequencing and Western blot analysis of related genes and proteins. First, we singled out related genes from all differentially expressed genes for heatmap analysis. As depicted in Figure 5A, tight junction genes (CLDN1, TJP1, and PECAM1) and anti-apoptosis genes (Bcl2) were up-regulated, while genes related to adhesion molecules (CD44, VCAM1, and ICAM1) and vascular permeability (AQP5) were downregulated in the LV-Apelin+ group. In contrast, genes related to adhesion molecules (VCAM1, ICAM1, and CA3) and apoptosis (Bax) were upregulated, while tight junction genes (EMP1, CLDN1, TJP1, and RAC1) were down-regulated in the LV-Apelin− group (Figure 5A). Second, we investigated relevant pathways through gene set enrichment analysis (GSEA), as described in a previous study [14]. Figure 5B illustrated that LV-Apelin+ upregulated the PI3K-Akt signaling and tight junction pathways and downregulated the NF-kappa B signaling and cell adhesion molecules’ pathways. Conversely, LV-Apelin− upregulated the apoptosis, cell adhesion molecules, and NF-kappa B signaling pathways, but downregulated the tight junction pathway (Figure 5B). At the protein level, we measured the levels of ZO-1 and occludin, which were closely associated with cell–cell tight junctions. In addition, the Western blot images (Figure 5C) and quantitative statistics (Figure 5D) revealed that the levels of ZO-1 and occludin increased in the LV-Apelin+ group, but decreased in the LV-Apelin− group.

### 2.6. The Effect on the Cerebrum in DR Mice Treated with LV-EGFP, LV-Apelin+, or LV-Apelin−

**Figure 6:**
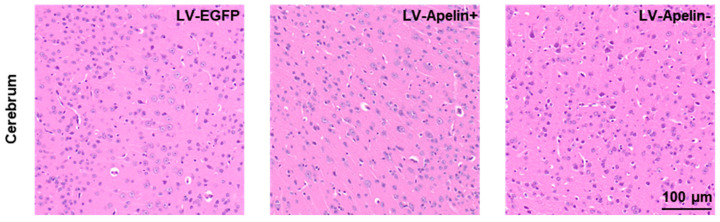
Effects on the cerebrum in DR mice treated with LV-EGFP, LV-Apelin+, or LV-Apelin−. Representative HE staining images revealed that the intravitreal injection of the LV had no obvious side effects on the cerebrum.

Given the proximity of the eyes and the cerebrum, we investigated the effect of the intravitreal injection of the LV on the cerebrum. In each group, HE staining revealed a few abnormalities in the cerebrum tissues (Figure 6), thereby indicating that the intravitreal injection of the LV exhibited satisfactory biosafety.

## 3. Discussion

In this study, we demonstrated that apelin plays a protective role for pericytes by reducing vascular leakage in the early stages of DR. To the best of our knowledge, this is the first study to reveal the effect of apelin on retinal pericytes and vascular permeability using lentiviral genetic modification technology in an in vivo model of DR. Importantly, we elucidated the relevant mechanism by which apelin can regulate the permeability of the retina by affecting the expression of pericytes. The results suggest that increasing the content or expression of apelin may constitute a potential treatment for vascular leakage in early DR.

One of the most significant symptoms of early DR is vessel leakage caused by increased vascular permeability. Tight junctions between endothelial cells play a crucial function in regulating vascular permeability [15]. More critically, pericyte dropout is an essential factor in destabilizing retinal vascular endothelial cells, which accelerates the progression of DR [5]. As revealed in previous studies, the loss of pericytes increased the endothelial permeability in the early stages of DR [16,17]. Therefore, preventing vascular leakage by reducing pericyte loss in early-stage DR may be an effective strategy to save the patient’s vision [18].

The apelin/APJ system plays an important role in vascular stabilization factors, including proliferation and permeability [19,20]. Recently, the definite genetic variant rs3115757 of the apelin gene was introduced to potentially affect the production of apelin in adipocytes. This functional variant may be associated with obesity features, insulin resistance indices, and the prevalence of type 2 diabetes mellitus [21]. According to previous studies, apelin can regulate the expression of cell adhesion molecules to improve vascular integrity and decrease permeability [13,22]. Our previous research proved that apelin does not only promote the development and stability of retinal blood vessels but also protects pericytes from hypoxia-induced apoptosis [11,12]. In this study, we compared the effects of the overexpression and knockdown of apelin on pericyte and retinal leakage in an early DR mouse model. Encouragingly, the overexpression of apelin significantly reduced the loss of pericytes and vascular leakage in early DR, whereas the knockdown of apelin had the opposite effect. These results were supported by subsequent mechanistic investigations.

The genes related to cell adhesion molecules were downregulated in response to apelin overexpression, whereas the cell–cell tight junctions and anti-apoptotic genes were up-regulated. However, the alteration of these genes was reversed in response to apelin knockdown. It is well known that apelin can reduce apoptosis via the PI3K-Akt signaling pathway, which is considered a canonical anti-apoptotic signaling pathway [23]. In our findings, this signaling pathway was up-regulated due to the overexpression of apelin, which is in line with a previous study [24]. In contrast, the knockdown of apelin up-regulated the apoptosis signaling pathway instead. It has been reported that the production of cell adhesion molecules was mediated by the NF-kappa B signaling pathway [25]. Our results demonstrate that apelin overexpression down-regulates the NF-kappa B and cell adhesion molecules’ signaling pathways, whereas apelin knockdown upregulates them. Simultaneously, the tight junction signaling pathway was up-regulated in the LV-Apelin+ group, which proves that apelin can decrease vascular permeability. In contrast, the tight junction signaling pathway was downregulated in the LV-Apelin− group. To further investigate the effect of apelin on retinal vascular permeability, we conducted a Western blot analysis to measure the content of retinal ZO-1 and occludin. As evident from the results, increased expressions of ZO-1 and occludin were found in the LV-Apelin+ group, while decreased expression were found in the LV-Apelin− group, which strongly suggests that apelin can stabilize vascular permeability. Finally, we demonstrated the biosafety of the intravitreal injection of the LV using pathological section staining.

Our study also has several limitations. First, we could not determine the exact efficiency of apelin overexpression and knockdown using lentiviral vectors, which was a general limitation of viral transfection technology. However, we roughly quantified the differential expression of apelin in each experimental group using retinal immunofluorescence staining and qRT-PCR analysis. Second, we did not observe the effect of apelin on other retinal cells such as endothelial cells and ganglion cells; this remains to be verified in vivo by future research.

## 4. Materials and Methods

### 4.1. Animals

Male C57BL/6J mice were obtained from Charles River Laboratories (Beijing, China) and housed in a 12 h dark and light cycle for a week. All animal protocols were approved by the Ethics Committee of Capital Medical University (AEEI-2022-078) and in compliance with the National Research Council’s Guide for the Care and Use of Laboratory Animals.

### 4.2. HFD/STZ-Induced Type 2 Diabetic Mouse Model

The type 2 diabetic mouse model was induced as previously described [26,27]. First, all animals were fed on HFD for four weeks, and remained on the diet until the end of the study. Subsequently, STZ (Sigma-Aldrich, St. Louis, MO, USA) was prepared in a pH 4.5 sodium citrate buffer and intraperitoneally injected at a dose of 100 mg/kg. The mice were randomly assigned to one of three groups: the LV-EGFP group (n = 23), the LV-Apelin+ group (n = 21), or the LV-Apelin− group (n = 21). In the meantime, six mice were randomly selected from each group for the measurement of body weight and blood glucose every two weeks. Two weeks after STZ injection, the mice with random blood glucose levels above 16.7 mmol/L were considered as mice with type 2 diabetes. Four weeks after the STZ injection, the LV was administered via intravitreal injection. Eight weeks later, all mice were euthanized and further experiments were proceeded.

### 4.3. Lentiviral Production

LV-EGFP (pLV[shRNA]-EGFP:T2A:Puro-U6>Scramble_shRNA), LV-Apelin+ (pLV[Exp]-Puro-EF1A>mApln[NM_013912.4]) and LV-Apelin− (pLV[shRNA]-Puro-U6>mApln[shRNA]) were packaged and titered following the manufacturer’s instructions (Haixingshengwu, Suzhou, China). Lentiviral production was performed as described in a previous study [28]. Briefly, the lentiviral vectors were added to 293T cells at a density of 70%; 8 h after transfection, the medium was replaced with fresh medium, and cell supernatants were collected after 48 h of culture. Subsequently, the supernatant was centrifuged at 1000× *g* for 10 min to remove residual cells and debris. Lentiviral solutions (109 TU/mL) were stored in 50 μL aliquots at −80 °C.

### 4.4. Immunofluorescence Staining of the Retina

Immunofluorescence staining of the retina was conducted with in accordance with a previously employed method [29]. To prepare retinal sections, the eyeballs were embedded in an optimum cutting temperature compound (Sakura 4583, Torrance, CA, USA) and quickly frozen in liquid nitrogen. Next, the eyes were sagittally cut into 10 μm sections through the cornea and parallel to the optic nerve in a cryostat (Leica CM1950, Berlin, Germany) at −20 °C. To prepare whole-mounted retinas, the eyeballs were fixed in 4% paraformaldehyde for 2 h at room temperature and then the retinas were dissected. For the immunofluorescence staining of retinal sections, these sections were fixed in 4% paraformaldehyde for 30 min and then incubated with 0.1% Triton X-100 for 10 min. Next, the sections were blocked with 10% goat serum for 1 h at room temperature and then incubated with primary antibody apelin (1:200, no. DF13350, Affinity Biosciences, Liyang, China) at 4 °C overnight. After several washes, the sections were incubated with Cy3-conjugated goat anti-rabbit secondary antibodies (1:200, no. S0011, Affinity) for 2 h at room temperature. For the immunofluorescence staining of retinal whole-mounts, these retinal whole-mounts were incubated with 10% goat serum in 0.1% Triton X-100 for 2 h at room temperature and then incubated with primary antibody NG2 (1:100, no. sc-53389, Santa Cruz Biotechnology, Dallas, TX, USA) and CD31 (1:100, no. 347526, Zen Bioscience, Chengdu, China) at 4 °C overnight. After several washes, the whole-mounts were incubated with DyLight 488-conjugated goat anti-mouse secondary antibody (1:200, no. E032210-01, EarthOx, San Francisco, CA, USA) and Cy3-conjugated goat anti-rabbit secondary antibody (1:200, no. S0011, Affinity) for 2 h at room temperature. The retinal sections and flat mounts were imaged using a confocal laser scanning microscope (CLSM) (A1, Nikon, Tokyo, Japan). ImageJ software was used to analyze the expression of apelin and NG2.

### 4.5. Quantitative Real-Time Polymerase Chain Reaction (qRT-PCR) Analysis

Total RNA was extracted using RNAeasy™ Animal RNA Isolation Kit with Spin Column (Beyotime Biotechnology, Shanghai, China). The following primers were used to study the expressions of mouse GAPDH and apelin: mouse GAPDH forward: 5′-CCACTGTGGGCCACTTATACC-3′; mouse GAPDH reverse: 5′-CAGCCTTAGCCGAGCATTG-3′; mouse apelin forward: 5′-GGAATTCGGGACCATGAATCTGAGGCTCTG-3′; and mouse apelin reverse: 5′-ACTTGGCGAGCCCTTCAATC-3′. The qRT-PCR analysis was conducted using BeyoFast™ SYBR Green One-Step qRT-PCR Kit (Beyotime Biotechnology, Shanghai, China) on the platform of a CFX96 Real-Time PCR Detection System (Bio-Rad, Hercules, CA, USA). The relative abundance of apelin was normalized by the level of GAPDH mRNA expression.

### 4.6. Retinal Leakage Analysis

Vascular leakage was analyzed by intravenous injection of 100 μL tetramethylrhodamine isothiocyanate–dextran (1.5 mg/mL, 65 kD-85 kD, Sigma-Aldrich) 40 min before the eye harvest [30]. First, the eyeballs were fixed in 4% paraformaldehyde for 2 h at room temperature. Next, eyes were enucleated and the retinas were dissected in PBS. Finally, the retinal flat mounts were imaged using a CLSM.

### 4.7. Transcriptome Sequencing Analysis

Collected retina tissues (one sample contained six eyes from three mice) were fast-frozen in liquid nitrogen and then sent to Gene Denovo Biotechnology Co., Ltd. (Guangzhou, China) for sample preparation and detection. The RNA sequencing analysis was performed according to the previously described experimental procedure [31]. Briefly, total RNA was extracted using TRIzol (Invitrogen, Carlsbad, CA, USA) and quality controlled by Agilent 2100 Bioanalyzer (Agilent Technologies, Palo Alto, CA, USA) according to the manufacturer’s prescribed protocol. Subsequently, mRNA was enriched by Oligo(dT) beads and then fragmented into short fragments using a fragmentation buffer. Immediately, the enriched mRNA was reverse transcribed into cDNA which was purified with a QiaQuick PCR extraction kit (Qiagen, Hilden, Germany). Next, the cDNA was ligated to the Illumina HiSeq2500 System for sequencing. A differential expression analysis of RNAs was performed using DESeq2 software among different groups. Genes with a log2 fold change > 2 and an adjusted *p* < 0.05 were deemed to be significantly differentially expressed.

### 4.8. Western Blot Analysis

The Western blot analysis was performed referring to the previous step [32]. Each group contained retina tissues of four eyes from two mice. To extract retinal total protein, the retinas were placed in a tissue lysate consisting of 100 μL RIPA and 1 μL PMSF for homogenizing. Subsequently, the homogenate was centrifuged at 10,000× *g* for 5 min and the supernatant was taken to detect the protein concentration using a BCA protein assay kit (Solarbio, Beijing, China). An equal dose of protein (20 μg) for each sample was loaded in a 4–15% Precast-Gel (Solarbio, Beijing, China). After electrophoresis, the protein was transferred onto a polyvinylidene difluoride membrane (Millipore, Billerica, MA, USA) using a wet transfer process. Next, the membrane was blocked with 5% nonfat dry milk for 2 h and then exposed to primary antibody ZO-1 (1:1000, no. 40-2300, Invitrogen), occludin (1:5000, no. 66378-1-lg, Proteintech, Chicago, IL, USA), or GAPDH (1:5000, no. ab8245, Abcam, Cambridge, UK) overnight at 4 °C. The following secondary antibody was HRP-conjugated goat anti-rabbit IgG (1:5000, no. ab97051, Abcam) and HRP-conjugated goat anti-mouse IgG (1:5000, no. ab205719, Abcam). After washing, the membrane was exposed to enhanced chemiluminescence (Millipore, Billerica, MA, USA), and the bands were detected using the MF-ChemiBIS system. ImageJ software was used to analyze the gray value of the bands. All experiments were performed in triplicate.

### 4.9. Hematoxylin-Eosin (HE) Staining of the Cerebrum

Cerebrums were fixed in 4% paraformaldehyde, embedded in paraffin, and cut into 5 μm sections in a coronal plane. After deparaffinization, HE staining was performed and observed under a light microscope.

### 4.10. Statistical Analysis

Statistical analysis was performed using Graphpad Prism 9.0.0 software. All graphs present mean ± SD. Comparisons were made using a two-tailed unpaired Student’s *t*-test for two groups or a one-way analysis of variance for multiple groups. *p* < 0.05 was considered statistically significant.

## 5. Conclusions

In conclusion, our study revealed that apelin protects the pericytes through the PI3K-Akt/Bcl2 signaling pathway and prevents the breakdown of the blood-retinal barrier through the NF-kappa B/Cell adhesion molecules and the tight junction signaling pathway in the early stages of DR. Our findings indicate that utilizing the protective properties of apelin might constitute a novel strategy in the early treatment of DR.

## Data Availability

The RNA-sequencing data for this study are available at the NCBI BioProject under accession PRJNA884176.

## References

[1] Groeneveld Y., Tavenier D., Blom J.W., Polak B.C.P. (2019). Incidence of sight-threatening diabetic retinopathy in people with Type 2 diabetes mellitus and numbers needed to screen: A systematic review. Diabet. Med..

[2] Cui Y., Zhang M., Zhang L., Zhang L., Kuang J., Zhang G., Liu Q., Guo H., Meng Q. (2019). Prevalence and risk factors for diabetic retinopathy in a cross-sectional population-based study from rural southern China: Dongguan Eye Study. BMJ Open.

[3] Arboleda-Velasquez J.F., Valdez C.N., Marko C.K., D’Amore P.A. (2015). From pathobiology to the targeting of pericytes for the treatment of diabetic retinopathy. Curr. Diabetes Rep..

[4] Caporarello N., D’Angeli F., Cambria M.T., Candido S., Giallongo C., Salmeri M., Lombardo C., Longo A., Giurdanella G., Anfuso C.D. (2019). Pericytes in Microvessels: From “Mural” Function to Brain and Retina Regeneration. Int. J. Mol. Sci..

[5] Park D.Y., Lee J., Kim J., Kim K., Hong S., Han S., Kubota Y., Augustin H.G., Ding L., Kim J.W. (2017). Plastic roles of pericytes in the blood-retinal barrier. Nat. Commun..

[6] Ferland-McCollough D., Slater S., Richard J., Reni C., Mangialardi G. (2017). Pericytes, an overlooked player in vascular pathobiology. Pharmacol. Ther..

[7] Uribesalgo I., Hoffmann D., Zhang Y., Kavirayani A., Lazovic J., Berta J., Novatchkova M., Pai T.P., Wimmer R.A., László V. (2019). Apelin inhibition prevents resistance and metastasis associated with anti-angiogenic therapy. EMBO Mol. Med..

[8] Chen K., Zhao X.L., Li L.B., Huang L.Y., Tang Z., Luo J., Yang L., Qin A.P., Hu F. (2020). miR-503/Apelin-12 mediates high glucose-induced microvascular endothelial cells injury via JNK and p38MAPK signaling pathway. Regen. Ther..

[9] Song B., Kim D., Nguyen N.H., Roy S. (2018). Inhibition of Diabetes-Induced Lysyl Oxidase Overexpression Prevents Retinal Vascular Lesions Associated With Diabetic Retinopathy. Investig. Ophthalmol. Vis. Sci..

[10] Kidoya H., Takakura N. (2012). Biology of the apelin-APJ axis in vascular formation. J. Biochem..

[11] Feng J., Chen L., Jiang Y., Tao Y. (2020). The Role of Apelin/APJ in a Mouse Model of Oxygen-induced Retinopathy. Investig. Ophthalmol. Vis. Sci..

[12] Chen L., Tao Y., Feng J., Jiang Y.R. (2015). Apelin Protects Primary Rat Retinal Pericytes from Chemical Hypoxia-Induced Apoptosis. J. Ophthalmol..

[13] Kidoya H., Naito H., Takakura N. (2010). Apelin induces enlarged and nonleaky blood vessels for functional recovery from ischemia. Blood.

[14] Subramanian A., Tamayo P., Mootha V.K., Mukherjee S., Ebert B.L., Gillette M.A., Paulovich A., Pomeroy S.L., Golub T.R., Lander E.S. (2005). Gene set enrichment analysis: A knowledge-based approach for interpreting genome-wide expression profiles. Proc. Natl. Acad. Sci. USA.

[15] Cong X., Kong W. (2020). Endothelial tight junctions and their regulatory signaling pathways in vascular homeostasis and disease. Cell Signal.

[16] Yun J.H. (2021). Interleukin-1β induces pericyte apoptosis via the NF-κB pathway in diabetic retinopathy. Biochem. Biophys. Res. Commun..

[17] Kim Y.H., Kim Y.S., Roh G.S., Choi W.S., Cho G.J. (2012). Resveratrol blocks diabetes-induced early vascular lesions and vascular endothelial growth factor induction in mouse retinas. Acta Ophthalmol..

[18] Stitt A.W., Curtis T.M., Chen M., Medina R.J., McKay G.J., Jenkins A., Gardiner T.A., Lyons T.J., Hammes H.P., Simó R. (2016). The progress in understanding and treatment of diabetic retinopathy. Prog. Retin. Eye Res..

[19] Mughal A., O’Rourke S.T. (2018). Vascular effects of apelin: Mechanisms and therapeutic potential. Pharmacol. Ther..

[20] Kasai A., Ishimaru Y., Higashino K., Kobayashi K., Yamamuro A., Yoshioka Y., Maeda S. (2013). Inhibition of apelin expression switches endothelial cells from proliferative to mature state in pathological retinal angiogenesis. Angiogenesis.

[21] Aboouf M.A., Hamdy N.M., Amin A.I., El-Mesallamy H.O. (2015). Genotype screening of APLN rs3115757 variant in Egyptian women population reveals an association with obesity and insulin resistance. Diabetes Res. Clin. Pract..

[22] Li B., Yin J., Chang J., Zhang J., Wang Y., Huang H., Wang W., Zeng X. (2021). Apelin/APJ relieve diabetic cardiomyopathy by reducing microvascular dysfunction. J. Endocrinol..

[23] Luo K., Long H., Xu B., Luo Y. (2015). Apelin attenuates postburn sepsis via a phosphatidylinositol 3-kinase/protein kinase B dependent mechanism: A randomized animal study. Int. J. Surg..

[24] Xie H., Yuan L.Q., Luo X.H., Huang J., Cui R.R., Guo L.J., Zhou H.D., Wu X.P., Liao E.Y. (2007). Apelin suppresses apoptosis of human osteoblasts. Apoptosis.

[25] Zhong L., Simard M.J., Huot J. (2018). Endothelial microRNAs regulating the NF-κB pathway and cell adhesion molecules during inflammation. FASEB J..

[26] Neumann U.H., Ho J.S.S., Chen S., Tam Y.Y.C., Cullis P.R., Kieffer T.J. (2017). Lipid nanoparticle delivery of glucagon receptor siRNA improves glucose homeostasis in mouse models of diabetes. Mol. Metab..

[27] Cheng Y., Yu X., Zhang J., Chang Y., Xue M., Li X., Lu Y., Li T., Meng Z., Su L. (2019). Pancreatic kallikrein protects against diabetic retinopathy in KK Cg-A(y)/J and high-fat diet/streptozotocin-induced mouse models of type 2 diabetes. Diabetologia.

[28] Zhuang P., Muraleedharan C.K., Xu S. (2017). Intraocular Delivery of miR-146 Inhibits Diabetes-Induced Retinal Functional Defects in Diabetic Rat Model. Investig. Ophthalmol. Vis. Sci..

[29] Laliberté A.M., MacPherson T.C., Micks T., Yan A., Hill K.A. (2011). Vision deficits precede structural losses in a mouse model of mitochondrial dysfunction and progressive retinal degeneration. Exp. Eye Res..

[30] Qin Y.J., Xiao K., Zhong Z., Zhao Y., Yu T., Sun X.F. (2022). LECT2 Ameliorates Blood-Retinal Barrier Impairment Secondary to Diabetes Via Activation of the Tie2/Akt/mTOR Signaling Pathway. Investig. Ophthalmol. Vis. Sci..

[31] Lee K., Choi J.O., Hwang A., Bae H.W., Kim C.Y. (2022). Ciliary Neurotrophic Factor Derived From Astrocytes Protects Retinal Ganglion Cells Through PI3K/AKT, JAK/STAT, and MAPK/ERK Pathways. Investig. Ophthalmol. Vis. Sci..

[32] Wang S., Li F., Ye T., Wang J., Lyu C., Qing S., Ding Z., Gao X., Jia R., Yu D. (2021). Macrophage-tumor chimeric exosomes accumulate in lymph node and tumor to activate the immune response and the tumor microenvironment. Sci. Transl. Med..

